# TP53 mutation detected in circulating exosomal DNA is associated with prognosis of patients with hepatocellular carcinoma

**DOI:** 10.1080/15384047.2022.2094666

**Published:** 2022-08-03

**Authors:** Yong Li, Junjun Wu, Enliang Li, Zhouqing Xiao, Jun Lei, Fan Zhou, Xiangbao Yin, Dandan Hu, Yilei Mao, Linquan Wu, Liao Wenjun

**Affiliations:** a Department of Hepatobiliary and Pancreatic Surgery, The Second Affiliated Hospital of Nanchang University, Nanchang, China; b Department of Hepatobiliary and Pancreatic Surgery, Jiangxi Provincial People’s Hospital, Nanchang, China; c Department of Hepatobiliary Surgery, Sun Yat-sen University Cancer Center, Guangzhou, China; d Department of Hepatobiliary Surgery, Peking Union Medical College, Beijing, China

**Keywords:** Liquid biopsy, exosomal DNA, hepatocellular carcinoma, droplet digital PCR, TP53 mutation

## Abstract

Exosome DNA (exoDNA) can be used for liquid biopsy. This study was the first to use droplet digital PCR (ddPCR) to detect tumor-specific mutations in exoDNA and to evaluate the prognosis of hepatocellular carcinoma (HCC) patients. 60 HCC patients were enrolled in the study. We used ddPCR to detect c.747 G > T mutation in TP53 gene. We analyzed the correlation between detectable mutation in exoDNA and clinicopathologic characteristics using Multivariate logistics regression analysis. We performed Cox regression to assess the correlation between mutation frequency (mutant droplets/total droplets, MD/TD) and prognostic. We found that 48 of 60 patients had c.747 G > T mutation in TP53 gene in exoDNA (80.0%). We found that detectable mutation in exoDNA and age were associated with microvascular invasion (MVI) (P < .01). The ROC curve analysis revealed that the best cutoff value of mutation frequency to predict MVI was 67% (sensitivity 48.15%, specificity 93.94%,), the corresponding AUC was 0.761 (95%CI, 0.640–0.866; P < .01). Furthermore, we found that patients suffered high-frequency mutation (>67%) had shorted median recurrence-free survival (RFS) with 63 days (range, 53–202 days), compared with 368 days (range, 51–576 days) for patients with low-frequency mutation (<67%) (HR:4.61; 95% CI, 1.70–12.48; P = 0 .003). We also found that high-frequency mutation was associated with poor prognosis though patients had better pathological characteristics, such as AFP (<400 ng/mL), Liver cirrhosis (Negative), Tumor thrombus (Negative), Tumor numbers (Single) and Post-operation TACE (Executed). We provided evidence that the mutations in exoDNA might be used to predict patients with poor RFS.

**Abbreviations:** TP53: Tumor protein p53; ExoDNA: Exosomal DNA; HCC: Hepatocellular carcinoma; ddPCR: Droplet digital Polymerase Chain Reaction (PCR); MD/TD: The ratio of mutant droplets/total droplets; AFP: Alpha-fetoprotein; MVI: Microvascular invasion; RFS: Recurrence-free survival.

## Background

Hepatocellular carcinoma (HCC) is currently the fifth most common malignancy with the third highest mortality rate in the world.^1^ Effective treatment and diagnostic strategies for HCC remain difficult problems. Although there are many treatments for HCC, including surgery, transcatheter arterial chemoembolization, and targeted drugs, the recurrence rate of HCC is high.^1,2^ Therefore, timely diagnosis and real-time intervention are required for effective diagnostic and therapeutic measures for HCC. Unfortunately, the diagnostic efficacy of traditional markers, such as AFP, was not satisfactory.^3^ In recent years, multiple studies have used circulating free DNA (cfDNA) from the blood of pancreatic and colorectal cancer patients to identify tumor-specific mutations as cancer markers.^4,5^ However, cfDNA originates from dead cells in damaged tissue or accumulates as a result of physiological cell turnover.^6,7^ This may lead to reduced sensitivity of cfDNA bio-markers and make the identification of cancer-specific mutations more challenging.

Exosomes are extracellular vesicles 40–150 nm in size that are rich in DNA, RNA, and proteins.^8^ Although exoDNA shows biological stability and has the potential for clinical application, few studies have examined exoDNA compared with the multiple studies already performed on exosomal RNA. Exosomes are secreted by living and dead cells, as well as cancer cells, which produce high numbers of exosomes.^8–10^ Recent studies have shown that circulating exoDNA can be used to identify cancer-specific mutations,^9,11,12^ which is of great significance for the diagnosis and treatment of HCC patients as well as prognosis assessment. Genetic mutations have been traditionally identified by genome sequencing of biopsy tumor fragments or cfDNA, but this represents only a small fraction of tumor heterogeneity because cfDNA is released by dead tumor cells.^6^ ExoDNA is more stable than cfDNA because of the lipid bilayer structure of exosomes,^13,14^ and exosomes contain larger DNA fragments, which is conducive to the detection of mutations. Additionally, because exosomes can be released from various cells in tumors, exoDNA in plasma may reflect tumor heterogeneity. Based on these advantages, increasing studies have been reported on tumor-specific mutations in circulating exoDNA, such as *EGFR* mutations in lung adenocarcinoma patients and *KRAS* mutations in pancreatic cancer patients.^9,11,15^ However, no studies have been reported on the detection of tumor-specific mutations in circulating exoDNA in HCC patients.

In this study, here we report the first investigation of the value and significance of detection of tumor-specific mutations in circulating exoDNA in HCC patients using droplet digital PCR (ddPCR). And we found that patients with high-frequency mutation are more likely to microvascular invasion and associated with poor prognosis.

## Results

### Patient characteristics

This study included 60 patients with primary HCC who were enrolled between October 2018 and August 2020 at the Second Affiliated Hospital of Nanchang University. The median age in this study was 56 years, with 40 (66.7%) males and 20 (33.3%) females. The proportion of HBV infection and cirrhosis was 58 (96.7%) and 33 (55.0%) respectively, 27 patients (45.0%) had Microvascular invasion, 8 patients (13.3%) had Tumor thrombus and 40 patients (33.3%) with preoperative AFP ≥400 ng/ml. The median of tumor size was 6.2 cm, with the liver function of all patients was Child A. In these patients, 48 (80.0%) had been detected TP53 mutation in circulating exoDNA. The clinical characteristics of these 60 patients are listed in Table 1. Additionally, we presented the detailed preoperative information of these patients in Supplementary Table S1.

**Table 1.** Clinical characteristics of patients in this study.

Clinical Characteristics	N = 60 (%)
Gender	
Female	20(33.3)
Male	40(66.7)
TP53 mutation	
Positive	48(80.0)
Negative	12(20.0)
HBV	
Positive	58(96.7)
Negative	2(3.3)
Cirrhosis	
Positive	33(55.0)
Negative	27(45.0)
Preoperative AFP(ng/ml)	
<400	20(33.3)
≥400	40(66.7)
Tumor thrombus	
Positive	8(13.3)
Negative	52(86.7)
Microvascular invasion	
Positive	27(45.0)
Negative	33(55.0)
Satellite nodules	
Positive	19(31.7)
Negative	41(68.3)
Postoperation TACE	
Executed	39(65.0)
Non-executed	21(35.0)

Abbreviations: TP53 mutation, tumor protein p53 mutation status in circulating Exosomal DNA; HBV, hepatitis B virus; AFP, alpha-fetoprotein.

### TP53 mutation in exoDNA and clinicopathologic characteristics correlation

The c.747 G > T mutation in TP53 had been identified as one of the hottest mutants in HCC patients.^16–18^ And our previous studies had confirmed the presence of TP53 mutations in circulating tumor DNA in HCC patients.^16^ Thus, these mutations might be detectable in exoDNA, which also contained abundant tumor cell-derived genetic information. We detected TP53 gene mutation status (c.747 G > T) in circulating exoDNA and presented the copy number of wild-type and mutant TP53 gene in all patients. We found that 48 of 60 patients had c.747 G > T mutations in TP53 gene in exoDNA (sensitivity, 80.0%). The high sensitivity may be due to the wide source and stability of exosomes. Multivariate logistics regression analysis revealed that TP53 mutation and age was associated with microvascular invasion (MVI) (P < .001), but were not significantly associated with other clinicopathological features such as Satellite nodules, tumor number, and AFP (Table 2) .

**Table 2.** Results of correlation multivariate regression analysis between patient characteristics and MVI.

patient characteristics	Odds Ratio	95%CI		P
		Lower	Upper	
intercept	0.73	0.00	448.02	0.93
TP53 mutations	1.07	1.02	1.13	<0.01
Age	0.85	0.76	0.96	<0.01
AFP	28.59	1.32	620.94	0.03
Satellite nodules	2.50	0.23	26.78	0.672
Cancer embolus	6.30	0.11	376.89	0.38
Sex	10.75	0.64	179.42	0.10
Size	1.37	0.94	2.00	0.10

Abbreviations: MVI: microvascular invasion; AFP: alpha-fetoprotein;

In order to explore the clinical application of exoDNA, we calculated and analyzed the ratio of mutant droplets/total droplets (MD/TD). We executed the ROC curve analysis and found that the best cutoff value of MD/TD to predict MVI was ≥67% (sensitivity 48.15%, specificity 93.94%), the corresponding AUC was 0.761 (95%CI [0.640–0.866], P < .01). The detailed results are shown in Supplementary Figure S1.

### TP53 mutation in exoDNA and prognosis correlation

**Figure 1.**
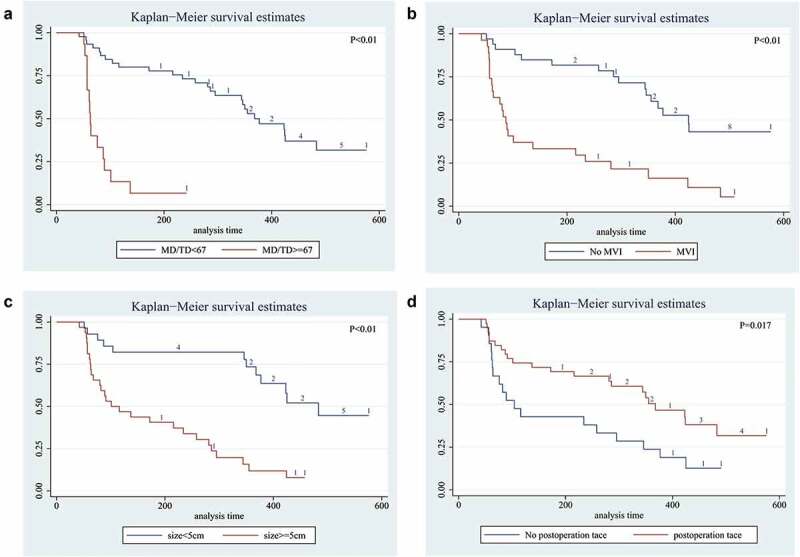
Long-term survival outcomes in HCC using Kaplan-Meier’s analysis:A. Survival time of mutation frequency in HCC. The results shown that the patients with MD/TD≥67 had shorter median survival time; B. MVI positive shown worse survival time; C. Tumor size >5 cm had worse result shorter survival time; D. Patient had not been postoperative tace had worse median survival than patients without satellite nodules. P value was assessed using the log-rank test.

We also analyzed whether TP53 mutation status in exoDNA could predict the prognosis of HCC patients who had undergone surgical treatment. We developed a regular and rigorous follow-up strategy of these 60 HCC patients up to 576 days. The results shown that patients suffered high-frequency mutations (MD/TD ≥67%) had shorted median RFS with 68 days (range, 53–202 days), compared with 368 days (range, 51–576 days) for patients with low-frequency mutations (MD/TD <67%). This result had statistically different (P < .01, log-rank test, Figure 1a). This result shown that patients in the group with high-frequency mutations were more likely to relapse than those in the group with low-frequency mutations. Similarly, the patients with pathological characteristics (such asMVI (+), tumor size >5 cm or postoperative tace (-)) had shorted median RFS (log-rank test, Figure 1).

**Figure 2.**
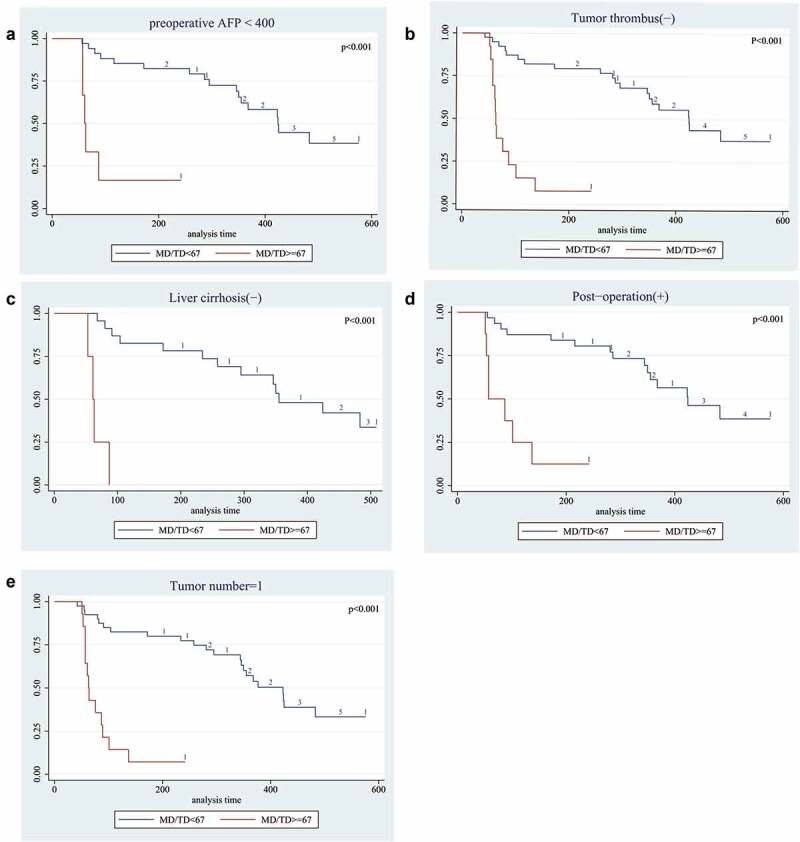
Long-term survival outcomes in HCC with mutation frequency using Kaplan-Meier’s analysis:A. Survival curve of mutation frequency in patients with AFP<400. The results shown that the patients with MD/TD≥67 had worse survival time; B. Survival curve of mutation frequency in patients with Tumor thrombus. The results shown that the patients with MD/TD≥67 had worse median survival time; C. Survival curve of mutation frequency in patients without liver cirrhosis. The results shown that patients with MD/TD≥67 had worse median survival time; D. Survival curve of mutation frequency in patients who treated by Post-operation TACE. The results shown that patients with high MD/TD had worse survival time; E. Survival curve of mutation frequency in patients with Single lesion. The results shown that MD/TD≥67 result had worse median survival time. P value was assessed using the log-rank test.

In addition, we also wonder whether there had correlation between mutation frequency and RFS in different pathological status. The results shown that the patients with high-frequency mutations (MD/TD ≥67%) had shorted RFS when preoperative AFP <400 ng/ml had been detected (median RFS:61 VS. 368 days, P < .01). In patients suffered single tumor, high-frequency mutations also predicted shorter survival times (median RFS:63 VS. 423 days, P < .01). Similar results could be seen in patients without tumor thrombus and liver cirrhosis (median RFS:63 VS. 424 days, P < .01; median RFS:61 VS. 355 days, P < .01). When patients executed TACE after operation, the status of high-frequency mutations also predicted shorter survival times (median RFS:57 VS. 424 days, P < .01). These results had been shown in Figure 2.

Based on multivariable Cox regression analysis, we found that high-frequency mutations in TP53 gene detected in exoDNA (MD/TD ≥67%) was associated with poor prognosis (hazard ratio [HR] = 4.61; 95% CI: 1.71–12.48; P = .003). In addition, we also found that MVI (Positive), postoperative tace (negative), and tumor size (>5 cm) were all independently associated with poor prognoses (Table 3). Furthermore, we also found when the patients with different status, such as AFP (<400 ng/mL), Liver cirrhosis (Negative), Tumor thrombus (Negative), Tumor numbers (Single) and TACE (Post-operation), high-frequency mutations was associated with poor prognosis (Table 4).

**Table 3.** Multivariate Cox’s Proportional Hazards Model Assessing Factors Associated with RFS.

Risks Factors	HR*	95%CI		P Vaule
		Lower	Upper	
Mutation frequency (MD/TD)				
<67	Referent			
≥67	4.61	1.70	12.48	0.003
Microvascular invasion				
(-)	Referent			
(+)	4.95	1.91	12.87	0.001
Size(cm)				
<5 cm	Referent			
≥5 cm	5.67	2.05	15.63	0.001
postoperation tace				
(-)	Referent			
(+)	0.42	0.21	0.86	0.018

*HRs greater than 1.0 indicates a higher risk of death

**Table 4.** Multivariate Cox’s Proportional Hazards Model Assessing Mutation frequency Associated with RFS in different Clinical characteristics.

status	Risks Factors(MD/TD)		HR*	95%CI		P Vaule
				Lower	Upper	
Preoperative AFP<400ng/L		<67	Referent			
		≥67	10.60	3.05	36.87	<0.001
No liver cirrhosis		<67	Referent			
		≥67	27.91	4.86	160.37	<0.001
No cancer embolus		<67	Referent			
		≥67	10.46	4.08	26.86	<0.001
Tumor number = 1		<67	Referent			
		≥67	9.51	3.83	23.57	<0.001
postoperation tace		<67	Referent			
		≥67	9.44	3.03	29.41	<0.001

Abbreviations: *HRs greater than 1.0 indicates a higher risk of death

## Discussion

ExoDNA is considered as stable cargo in exosomes and contained the mutations derived from cancers.^19^ These mutations that detected in random exoDNA fragments spanning multiple chromosomes and could be mimicked as “peripheral reservoir”.^20^ Recently, exosomal DNA had been considered as one of the most prospective liquid biopsy in oncology research.^21^ Many studies had found that the correlation between exoDNA and clinical outcomes in several cancers.^22^ However, no studies thus far tried to explore its application value in HCC patients. Therefore, we used ddPCR to detect TP53 mutation in circulating exoDNA of HCC patients and explored the possibility of circulating exoDNA as a new noninvasive liquid biopsy method in prognosis of HCC.

In our study,80.0% patients had been detected the TP53 mutations in exoDNA. The detection efficiency was immensely higher than that was used in cfDNA.^16^This result should be attributed to longer DNA fragments which were protected by exosomes in peripheral circulation. The total number of circulating DNA fragments <1.0%,^23,24^ and this made traditional technology difficult to detect. This was reason why cfDNA was considered as poor robustness using for HCC diagnosis.^25^ Targeted sequencing of circulating exoDNA had been applied to a variety of tumors. For example, Allenson et al. detected KRAS mutations in 66.7% (22/33), 80% (12/15) and 85% (17/20) of patients with localized, locally advanced and metastatic pancreatic cancer, respectively.^9^ It was similar to our sensitivity detection results.

In addition, our results shown that TP53 mutation in exoDNA was associated with MVI. It is easy to understand because the exosome secretion could be increased when microvascular invasion was presented. That means we can use the detectable tumor-associated mutations in exoDNA to predict vascular invasion status in patients indirectly. Wang et al. reported that circulating tumor DNA correlated with MVI and could use to predict tumor recurrence of HCC,^17^ this results were similar to ours. Futhermore, our results shown that there had no significantly correlation between mutations in exoDNA and other clinicopathological features such as Satellite nodules, tumor number, and AFP. It confirmed that the relevance between detectable tumor-associated mutations in exoDNA and MVI was uniqueness. This indicated that exoDNA had wide application prospect.

The greatest contribution of our study was that we found the correlation between exoDNA and poor prognosis in HCC patients. In our study, we found that the patients with high-frequency mutations in exoDNA had poor prognosis compared with patients with low-frequency mutations (P < .01), even with good clinicopathologic characteristics. Furthermore, although patients suffered single tumor, or without tumor thrombus and liver cirrhosis, the prognosis was poor if high-frequency mutations in exoDNA was existed. That means, the frequency mutations detected in exoDNA might be an independent risk factor for prognosis in HCC. This phenomenon might be the result for the burden of tumor. Normally, exoDNA was DNA fragments originating from either normal physiologic tissues or unnormal pathological tissues as a result of DNA accumulation.^10^ The equilibrium would be broken if the tumor-derived exosomes secretion was increased, and this would be reflected in the detection of high-frequency mutations in exoDNA. This is the reason why patients with high-frequency mutations in exoDNA would suffer poor prognosis.

In order to improve the detection efficiency of mutations in exoDNA, we used ddPCR described by Huggett JF.^26^ This method could provide higher sensitivity and precision for discrimination of rare mutant variants in exoDNA, and was extensive used.^27^

We anticipated that exoDNA would provide beneficial information for the prognosis of personalized HCC therapy. The detection of circulating exoDNA might enable further development of precision medical technologies to realize prognosis evaluation of HCC and to customize personalized treatment strategies.

## Methods

### Sample collection

A total of 60 patients with primary HCC at the Second Affiliated Hospital of Nanchang University were enrolled between October 2018 and August 2020. All patients agreed to the analysis of exoDNA mutations in collected blood before operation. Pathology was used to diagnose HCC. HCC patients were eligible if they agreed to have their blood collected for detection of exoDNA before surgery. None of the HCC patients received preoperative cancer-related therapies. Written informed consent was obtained from all patients enrolled in the study. Blood samples were collected in EDTA tubes, and within 1 h, samples were centrifuged at 3000 × g for 20 min. Serum samples were stored at −80°C. This study was approved by the Ethics Review committee of the Second Affiliated Hospital of Nanchang University.

### Exosome extraction and exoDNA extraction

Exosomes were extracted from serum samples using the ExoQuick™ kit (System Bioscience, CA, USA) following the manufacturer’s instructions. We examined the exosomes by electron microscopy and detection of the exosome-specific protein CD63. Protein expression of CD63 was assessed by western blotting and three experiments were repeated to verify the presence of CD63. ExoDNA was extracted from exosomes using the ExoDNA™ Extraction kit (BioVision Inc, SF, USA) following the manufacturer’s instructions. We used this method to extract exosome DNA and the presence of DNA was confirmed in all samples.

### Study design and droplet digital PCR (ddPCR)

DdPCR (also known as single-molecule PCR) generally consists of two steps: PCR amplification and fluorescence signal analysis. Our experimental procedures followed the guidelines proposed for reporting digital PCR data.^26^ The PCR primers and probe sequences were designed using Primer Premier 5.0 software. Each PCR reaction contained 10 μl ddPCR Supermix for Probes (Bio-Rad, USA), 3.6 μl of primer (Sangon Company, China), 1 μl of probe (Sangon Company), 2 μl of template DNA from exoDNA and 3.4 μl of ddH2O to give a total volume of 20 μl. The PCR conditions were 96°C for 10 min; 40 cycles of 94°C for 30 s and 60°C for 60s, with a final incubation at 98°C for 10 min.

### Preparation and analysis of droplets

We added a new DG8 Cartridge (BioAssay Systems, CA, USA) into the holder and added 20 μL of the samples into eight holes in the middle row. Next, 70 μL drop-generated Oil (DG Oil) was added into the bottom row of eight cartridges in the same chamber. The gasket was covered and the holes on both sides were firmly hooked. The holder was placed in the QX200 droplet generator (Bio-Rad, CA, USA) for generation of droplets, which generally took about 2 min to complete. Droplets were produced in the top row of cartridges and subsequently transferred into a 96-well plate. The plate was placed on a heat-sealing instrument and covered with a heat-sealing membrane for sealing. The QX200 Droplet analyzer was used for droplet analysis and detection, and the data were uploaded to the computer for final analysis. After the reactions were completed, the threshold line was adjusted to the appropriate position according to the specific reaction conditions for result interpretation. The 5’ primer ends of mutant and wild-type genes were labeled with the fluorescent dyes FAM (blue) and VIC (green), respectively, to assess the mutation status of the genotyped PCR product with the QX200 Droplet analyzer. Red fluorescence indicated that FAM and VIC were simultaneously detected.

## Statistical analysis

We used Stata software (version 16.0; Stata Corporation LP; College Station, TX, USA) to perform statistical analyses. Logistics regression analysis was used to assess the correlation between mutation status and the clinical and pathologic characteristics. Recurrence-free survival (calculated as the time from operation to tumor recurrence) curves were plotted according to Kaplan–Meier method and assessed using the log-rank test. Independent predictors of RFS were determined by Cox proportional hazard regression. Results were considered statistically significant if the P-value was less than 0.05.

## Conclusions

Our results provided evidence that the mutations in exoDNA might be used to predict patients with shorter recurrence-free survival.

## Supplementary Material

Supplemental Material
