# Supplementary material for: TP53 mutation detected in circulating exosomal DNA is associated with prognosis of patients with hepatocellular carcinoma
Source: Cancer Biol Ther. 2022 Aug 3;23(1):439–45. doi: 10.1080/15384047.2022.2094666 (PMC9354767; doi:10.1080/15384047.2022.2094666)
Supplement: Supplemental Material [file KCBT_A_2094666_SM0496.zip › 5Supplementary Table 1.doc]

**Supplementary Table1: Detailed information of HCC patients**

| **Subject** | **Sex** | **Age** | **Albumin**  **（g/L）** | **Tbil**  **(umol/l)** | **Afp**  **(ng/ml)** | **Pt**  **(s)** | **Cirrhosis** | **Wild**  **type** | [**mutant**](javascript:;) | **Mvi** | [**mutation**](javascript:;)  [**frequency**](javascript:;) | **Follow**  **time** | **Relapse**  **Status** | **Tumor**  **Thrombus** | **Mvi**  **grade** | **Tumor**  **number** | [**maximum**](javascript:;)  [**diameter**](javascript:;)**(cm)** | **Size**  **（cm）** | **Satellite**  **nodules** | **Postoperative**  **tace** | **Histological**  **grade** | **INR** | **Ast**  **(U/L)** | **Alt**  **(U/L)** | **Hbv** | **C-P**  **Class** |
| --- | --- | --- | --- | --- | --- | --- | --- | --- | --- | --- | --- | --- | --- | --- | --- | --- | --- | --- | --- | --- | --- | --- | --- | --- | --- | --- |
| p1 | M | 45 | 37 | 21 | 347 | 14.9 | Y | 0 | 0 | 0 | 0 | 400 | N | N | M0 | 1 | ＜5cm | 4.5*4.3.5 | N | N | Middle | 1.29 | 46 | 39 | Y | A |
| p2 | F | 74 | 33 | 24 | 495 | 12.8 | Y | 0 | 0 | 0 | 0 | 435 | N | N | M0 | 2 | ＞5cm | 1.5*1.7*1.2、 6.1*3.2*2.5 | N | N | Middle | 1.10 | 49 | 27 | Y | A |
| p3 | F | 55 | 33 | 15 | ＞1000 | 12.3 | Y | 0 | 0 | 0 | 0 | 344 | Y | Y | M0 | 1 | ＞5cm | 11*8*4 | Y | Y | Middle | 1.06 | 48 | 21 | Y | A |
| p4 | M | 56 | 45 | 10 | ＞1000 | 10.6 | N | 0 | 0 | 0 | 0 | 104 | Y | N | M0 | 1 | ＜5cm | 2*1.5*1 | N | N | Middle | 0.91 | 33 | 19 | Y | A |
| p5 | F | 57 | 40 | 16 | 53.7 | 11.2 | N | 0 | 1 | 0 | 0 | 68 | Y | N | M0 | 3 | ＞5cm | 13*5.5*4.5 | N | Y | Middle | 0.97 | 74 | 63 | Y | A |
| p6 | M | 49 | 41 | 18 | 36.5 | 11.8 | N | 0 | 0 | 0 | 0 | 355 | Y | N | M0 | 1 | ＞5cm | 9*9*7 | Y | Y | Middle | 1.02 | 41 | 41 | Y | A |
| p7 | F | 48 | 40 | 11 | ＜1.3 | 13.0 | Y | 0 | 0 | 0 | 0 | 576 | N | N | M0 | 1 | ＜5cm | 2.5*2.4*1.8 | N | Y | Middle | 1.09 | 36 | 26 | Y | A |
| p8 | M | 39 | 37 | 19 | 31.1 | 12.6 | N | 0 | 0 | 1 | 0 | 91 | Y | Y | M2 | 1 | ＞5cm | 13*11*10 | Y | Y | Middle | 1.09 | 149 | 41 | Y | A |
| p9 | M | 32 | 32 | 14 | 35.7 | 11.5 | N | 1 | 1 | 1 | 40 | 80 | Y | N | M2 | 1 | ＞5cm | 7.3*6*5 | N | Y | Middle | 0.99 | 31 | 51 | Y | A |
| p10 | F | 77 | 47 | 25 | ＜1.3 | 12.5 | N | 1 | 1 | 0 | 45.5 | 493 | N | N | M0 | 1 | ＜5cm | 1.5*1*1 | N | Y | Middle | 1.05 | 25 | 15 | Y | A |
| p11 | M | 56 | 40 | 28 | 3.7 | 12.0 | Y | 1 | 1 | 0 | 50 | 250 | N | N | M0 | 1 | ＜5cm | 2*2*1.5 | N | Y | Middle | 1.04 | 27 | 15 | Y | A |
| p12 | M | 43 | 45 | 19 | 41.7 | 11.1 | N | 1 | 1 | 1 | 50 | 483 | Y | N | M1 | 1 | ＜5cm | 4*4.2.5 | N | 2次 | Middle | 0.96 | 70 | 54 | Y | A |
| p13 | M | 36 | 47 | 24 | 28 | 12.4 | N | 1 | 1 | 1 | 50 | 350 | Y | N | M1 | 1 | ＜5cm | 2.8*2.5*2 | Y | Y | Middle | 1.07 | 31 | 39 | Y | A |
| p14 | F | 50 | 40 | 17 | 304 | 11.1 | Y | 1 | 1 | 0 | 50 | 530 | N | N | M0 | 1 | ＜5cm | 2.5*2*2 | N | Y | Middle | 0.96 | 20 | 16 | Y | A |
| p15 | M | 64 | 38 | 17 | 3.8 | 12.0 | Y | 1 | 1 | 0 | 50 | 368 | Y | N | M0 | 1 | ＜5cm | 2.5*2.2*2 | N | Y | Middle | 1.04 | 33 | 21 | Y | A |
| p16 | M | 40 | 32 | 10 | ＞1000 | 12.2 | Y | 1 | 1 | 1 | 50 | 55 | Y | Y | M2 | 1 | ＞5cm | 8.5*7.5*7 | N | Y | Low | 1.04 | 33 | 35 | Y | A |
| p17 | M | 62 | 43 | 10 | 3.6 | 11.4 | N | 1 | 1 | 1 | 50 | 509 | N | N | M1 | 1 | ＜5cm | 2*2*1.3 | N | Y | Middle | 0.98 | 34 | 30 | Y | A |
| p18 | M | 74 | 39 | 12 | 3.2 | 12.6 | N | 1 | 1 | 0 | 50 | 346 | Y | N | M0 | 1 | ＜5cm | 4.5*4*3.8 | N | N | High | 1.09 | 23 | 22 | Y | A |
| p19 | M | 65 | 36 | 11 | 11.3 | 13.0 | Y | 1 | 1 | 0 | 50 | 286 | Y | N | M0 | 2 | ＞5cm | 7*5.5*5 | N | Y | Middle | 1.13 | 36 | 25 | Y | A |
| p20 | M | 52 | 40 | 17 | ＞1000 | 11.9 | N | 1 | 1 | 1 | 50 | 313 | N | N | M1 | 1 | ＜5cm | 2.5*2.5*2.3 | N | Y | Middle | 1.03 | 62 | 87 | Y | A |
| p21 | M | 39 | 40 | 17 | 1.4 | 12.4 | Y | 1 | 1 | 0 | 50 | 446 | N | N | M0 | 1 | ＜5cm | 3*3*1.5 | N | Y | Middle | 1.07 | 25 | 21 | Y | A |
| p22 | M | 80 | 43 | 6.9 | 52.8 | 11.0 | N | 1 | 1 | 0 | 50 | 491 | N | N | M0 | 1 | ＜5cm | 4.5*4.5*2.6 | N | N | Middle | 0.95 | 26 | 28 | N | A |
| p23 | F | 65 | 43 | 22 | 432.8 | 11.8 | Y | 1 | 1 | 0 | 50 | 377 | Y | Y | M0 | 1 | ＜5cm | 1.5*1.2*1 | N | N | Middle | 1.02 | 43 | 22 | Y | A |
| p24 | M | 63 | 46 | 17 | 2.4 | 10.9 | N | 1 | 1 | 0 | 50 | 458 | N | N | M0 | 1 | ＞5cm | 6.5*5*5 | N | Y | Middle | 0.91 | 24 | 23 | Y | A |
| p25 | F | 56 | 44 | 21 | 20.4 | 11.5 | N | 1 | 1 | 0 | 55.6 | 295 | Y | N | M0 | 1 | ＞5cm | 10.5*7.5*7.5 | N | N | Middle | 0.99 | 34 | 34 | Y | A |
| p26 | M | 43 | 40 | 15 | 38 | 10.8 | Y | 1 | 1 | 0 | 60 | 400 | N | N | M0 | 1 | ＜5cm | 2.5*2*2 | N | Y | Middle | 1.06 | 42 | 28 | Y | A |
| p27 | F | 46 | 44 | 14 | 3.5 | 10.0 | Y | 1 | 1 | 1 | 60 | 56 | Y | N | M1 | 1 | ＞5cm | 11*4*1 | N | N | Middle | 0.86 | 62 | 15 | Y | A |
| p28 | M | 56 | 36 | 16 | ＞1000 | 11.3 | Y | 1 | 1 | 1 | 60 | 42 | Y | N | M2 | 1 | ＞5cm | 11*11*7 | Y | N | Middle | 0.96 | 82 | 24 | Y | A |
| p29 | M | 75 | 39 | 14 | 52.9 | 11.6 | Y | 1 | 1 | 0 | 60 | 116 | Y | N | M0 | 2 | ＞5cm | 8*4.5*4、2*1.5*1.5 | N | N | Middle | 1.00 | 33 | 28 | Y | A |
| p30 | M | 75 | 37 | 16 | ＜1.3 | 10.4 | N | 1 | 1 | 0 | 62.5 | 172 | Y | N | M0 | 1 | ＞5cm | 8.5*6.5*5 | N | Y | Middle | 0.90 | 32 | 16 | Y | A |
| p31 | M | 58 | 33 | 24 | 13.8 | 13.8 | N | 1 | 1 | 1 | 67 | 61 | Y | N | M2 | 1 | ＞5cm | 12*9*9 | Y | N | Middle | 1.16 | 39 | 61 | Y | A |
| p32 | M | 42 | 45 | 29 | ＞1000 | 11.5 | N | 1 | 1 | 1 | 75 | 53 | Y | N | M1 | 1 | ＞5cm | 10.5*7.5*4 | N | Y | Middle | 0.99 | 27 | 31 | Y | A |
| p33 | M | 66 | 40 | 8 | 277 | 12.6 | Y | 1 | 1 | 1 | 75 | 57 | Y | N | M1 | 1 | ＞5cm | 10*10*4.5 | Y | Y | Middle | 1.09 | 46 | 41 | Y | A |
| p34 | F | 58 | 30 | 42 | ＞1000 | 15.2 | Y | 1 | 1 | 0 | 80 | 51 | Y | N | M0 | 1 | ＜5cm | 4.5*4*3.5 | N | Y | Middle | 1.34 | 59 | 32 | Y | A |
| p35 | M | 51 | 43 | 12 | 16.3 | 11.1 | N | 0 | 1 | 1 | 100 | 87 | Y | N | M1 | 1 | ＜5cm | 4.5*3.5*3 | N | Y | Low | 0.96 | 16 | 11 | Y | A |
| p36 | M | 48 | 43 | 22 | 99.5 | 12.6 | Y | 1 | 1 | 1 | 100 | 242 | N | N | M1 | 1 | ＜5cm | 1.8*1.5*1.5 | N | Y | Middle | 1.09 | 39 | 45 | Y | A |
| p37 | M | 58 | 30 | 12 | ＞1000 | 13.4 | Y | 0 | 1 | 1 | 100 | 57 | Y | Y | M1 | 1 | ＞5cm | 15.5*10.5*10 | Y | Y | Low | 1.16 | 39 | 21 | Y | A |
| p38 | M | 57 | 41 | 13 | ＞1000 | 10.5 | Y | 1 | 1 | 1 | 100 | 137 | Y | N | M1 | 1 | ＞5cm | 8*7.5*7 | Y | Y | Middle | 0.90 | 33 | 21 | Y | A |
| p39 | M | 49 | 40 | 44 | 131 | 12.7 | Y | 1 | 1 | 1 | 100 | 57 | Y | N | M1 | 1 | ＜5cm | 3.7*3.0*1.2 | N | N | Middle | 1.10 | 29 | 7.5 | Y | A |
| p40 | F | 42 | 30 | 27 | ＞1000 | 11.9 | Y | 1 | 1 | 1 | 100 | 101 | Y | N | M1 | 1 | ＞5cm | 17*14*10.5 | Y | Y | Middle | 1.03 | 225 | 112 | Y | A |
| p41 | M | 57 | 36 | 20 | 358 | 11.2 | Y | 0 | 0 | 0 | 0 | 425 | Y | 0 | M0 | 1 | <5cm | 2.3*1.5*1.8 | N | N | Middle | 1.21 | 87 | 39 | Y | A |
| p42 | F | 55 | 33 | 30 | >1000 | 14.1 | N | 0 | 0 | 1 | 0 | 234 | Y | 1 | M1 | 1 | >5cm | 6.3*3.5*5 | Y | N | Middle | 0.94 | 74 | 31 | Y | A |
| p43 | F | 52 | 35 | 29 | 42.3 | 10.7 | N | 0 | 0 | 0 | 0 | 206 | N | 0 | M0 | 1 | >5cm | 7.2*5.3*3 | Y | Y | Middle | 1.14 | 98 | 47 | Y | A |
| p44 | F | 44 | 46 | 43 | 25.8 | 14.4 | Y | 0 | 0 | 0 | 0 | 356 | N | 0 | M0 | 1 | <5cm | 4.2*3.2*2 | N | Y | Middle | 0.93 | 74 | 46 | Y | A |
| p45 | M | 56 | 31 | 20 | 22.8 | 14.5 | N | 1 | 1 | 0 | 42.5 | 292 | N | 0 | M0 | 1 | >5cm | 6.1*2.3.3.6 | Y | Y | Middle | 1.13 | 125 | 20 | Y | A |
| p46 | M | 48 | 30 | 26 | 20.2 | 12.9 | N | 1 | 1 | 0 | 50 | 364 | N | 0 | M0 | 1 | <5cm | 4.5*3.4*3 | N | Y | Middle | 0.99 | 72 | 30 | Y | A |
| p47 | M | 42 | 40 | 43 | 252.3 | 10.7 | Y | 1 | 1 | 1 | 50 | 423 | Y | 0 | M1 | 1 | <5cm | 4.7*4*3.8 | N | Y | Middle | 1.24 | 33 | 37 | Y | A |
| p48 | M | 53 | 31 | 16 | >1000 | 12.6 | Y | 1 | 1 | 1 | 50 | 216 | Y | 1 | M2 | 2 | >5cm | 8*6.2*7 | Y | Y | Low | 1.18 | 27 | 16 | Y | A |
| p49 | F | 65 | 39 | 38 | 3.3 | 12.1 | N | 1 | 1 | 0 | 50 | 463 | N | 0 | M0 | 1 | <5cm | 2*1.8*1.5 | N | Y | Middle | 1.02 | 71 | 54 | N | A |
| p50 | M | 58 | 30 | 24 | 536.4 | 11.7 | Y | 1 | 1 | 1 | 50 | 281 | Y | 0 | M1 | 1 | >5cm | 9.4*8.7*7.3 | N | Y | Middle | 0.96 | 38 | 30 | Y | A |
| p51 | M | 48 | 46 | 23 | 32.6 | 11.2 | N | 1 | 1 | 0 | 50 | 493 | N | 0 | M0 | 1 | <5cm | 2.2*1.6*1.3 | N | Y | Middle | 0.92 | 52 | 15 | Y | A |
| p52 | F | 64 | 31 | 23 | 220.4 | 11.5 | N | 1 | 1 | 0 | 50 | 424 | Y | 0 | M0 | 1 | >5cm | 7.3*6.4*7.2 | N | Y | Middle | 0.96 | 117 | 18 | Y | A |
| p53 | F | 45 | 43 | 37 | 20.3 | 11.1 | Y | 1 | 1 | 0 | 60 | 285 | N | 0 | M0 | 1 | <5cm | 2.6*3.3*1.9 | N | Y | Middle | 1.23 | 82 | 42 | Y | A |
| p54 | F | 61 | 34 | 21 | >1000 | 14.4 | Y | 1 | 1 | 1 | 60 | 82 | Y | 0 | M2 | 1 | >5cm | 6.8*8.4*7.5 | Y | N | Middle | 1.08 | 73 | 23 | Y | A |
| p55 | F | 66 | 36 | 26 | <1.3 | 13.3 | N | 1 | 1 | 0 | 58.5 | 258 | Y | 0 | M0 | 1 | >5cm | 9.7*8.4*6.4 | N | N | Middle | 1.18 | 32 | 40 | Y | A |
| p56 | M | 57 | 33 | 9 | 13.5 | 12.2 | N | 1 | 1 | 0 | 67 | 63 | Y | 0 | M0 | 1 | >5cm | 7.4*6.8*4.8 | Y | N | Middle | 1.05 | 80 | 26 | Y | A |
| p57 | M | 51 | 35 | 38 | 628.3 | 12.8 | Y | 0 | 1 | 1 | 75.6 | 62 | Y | 0 | M2 | 2 | >5cm | 8.8*6.7*6.2 | Y | N | Middle | 0.95 | 98 | 33 | Y | A |
| p58 | F | 58 | 36 | 30 | >1000 | 12.7 | Y | 1 | 1 | 1 | 89.5 | 76 | Y | 0 | M1 | 1 | <5cm | 4.6*3.8*3.4 | N | N | Middle | 1.11 | 94 | 21 | Y | A |
| p59 | M | 57 | 39 | 43 | >1000 | 12.5 | Y | 1 | 1 | 1 | 100 | 89 | Y | 1 | M2 | 1 | >5cm | 10*9.4*5 | Y | N | Middle | 1.14 | 114 | 36 | Y | A |
| p60 | M | 46 | 32 | 15 | 728.3 | 14.2 | Y | 1 | 1 | 1 | 100 | 64 | Y | 0 | M1 | 1 | >5cm | 7.4*6.9*5.2 | Y | N | Middle | 0.92 | 145 | 18 | Y | A |

Abbreviations: M: M; F: F; HBV, hepatitis B virus; HCV, hepatitis C virus; MVI: microvascular invasion; AFP, alpha-fetoprotein; ALT, alanine aminotransferase; AST, aspartate aminotransferase; TBIL: total bilirubin; PT: prothrombin time; C-P Class: Child-Pugh Class; Y: Yes; N, No.
