# Supplementary material for: TP53 mutation detected in circulating exosomal DNA is associated with prognosis of patients with hepatocellular carcinoma
Source: Cancer Biol Ther. 2022 Aug 3;23(1):439–45. doi: 10.1080/15384047.2022.2094666 (PMC9354767; doi:10.1080/15384047.2022.2094666)
Supplement: Supplemental Material [file KCBT_A_2094666_SM0496.zip › Supplementary Table 2.doc]

**Supplementary Table 2: Detailed information of the preoperative status of HCC patients**

| **Subjects** | **HBV/HCV** | **Cirrhosis** | **AFP**  **(ng/ml)** | **ALT**  **(U/L)** | **AST**  **(U/L)** | **Albumin**  **(g/L)** | **TBIL**  **(μmol/L)** | **PT**  **(s)** | **C-P Class** | **MVI** | **Tumor size**  **≥5cm** | **Tumor**  **thrombus** | **Tumor**  **number** |
| --- | --- | --- | --- | --- | --- | --- | --- | --- | --- | --- | --- | --- | --- |
| **P1** | HBV | Y | 3.7 | 15 | 27 | 40 | 28 | 12.0 | A | N | Y | N | 1 |
| **P2** | HBV | Y | 16.3 | 11 | 16 | 43 | 12 | 11.1 | A | Y | N | N | 1 |
| **P3** | HBV | N | 41.7 | 54 | 70 | 45 | 19 | 11.1 | A | Y | N | N | 1 |
| **P4** | HBV | N | 28 | 39 | 31 | 47 | 24 | 12.4 | A | Y | N | N | 1 |
| **P5** | HBV | N | 304 | 16 | 20 | 40 | 17 | 11.1 | A | N | N | N | 1 |
| **P6** | HBV | Y | 3.8 | 21 | 33 | 38 | 17 | 12.0 | A | N | N | N | 1 |
| **P7** | HBV | Y | 347 | 39 | 46 | 37 | 21 | 14.9 | A | N | N | N | 1 |
| **P8** | HBV | Y | 495 | 27 | 49 | 33 | 24 | 12.8 | A | N | N | N | 2 |
| **P9** | HBV | Y | ＞1000 | 21 | 48 | 33 | 15 | 12.3 | A | N | Y | Y | 1 |
| **P10** | HBV | N | ＞1000 | 19 | 33 | 45 | 10 | 10.6 | A | N | N | N | 1 |
| **P11** | HBV | N | 35.7 | 51 | 31 | 32 | 14 | 11.5 | A | Y | Y | N | 1 |
| **P12** | HBV | Y | ＞1000 | 24 | 82 | 36 | 16 | 11.3 | A | Y | N | N | 1 |
| **P13** | HBV | N | 53.7 | 63 | 74 | 40 | 16 | 11.2 | A | N | Y | N | 2 |
| **P14** | HBV | N | 36.5 | 41 | 41 | 41 | 18 | 11.8 | A | N | N | N | 1 |
| **P15** | HBV | Y | ＞1000 | 35 | 33 | 32 | 10 | 12.2 | A | Y | Y | Y | 1 |
| **P16** | HBV | Y | ＜1.3 | 26 | 36 | 40 | 11 | 13.0 | A | N | N | N | 1 |
| **P17** | HBV | N | ＞1000 | 21 | 39 | 30 | 12 | 13.4 | A | Y | Y | Y | 1 |
| **P18** | HBV | Y | 3.6 | 30 | 34 | 43 | 10 | 11.4 | A | Y | N | N | 1 |
| **P19** | HBV | N | 3.2 | 22 | 23 | 39 | 12 | 12.6 | A | N | N | N | 1 |
| **P20** | HBV | Y | 13.8 | 61 | 39 | 33 | 24 | 13.8 | A | Y | Y | N | 1 |
| **P21** | HBV | Y | 11.3 | 25 | 36 | 36 | 11 | 13.0 | A | N | Y | N | 2 |
| **P22** | HBV | N | ＞1000 | 31 | 27 | 45 | 29 | 11.5 | A | Y | Y | N | 1 |
| **P23** | HBV | N | ＞1000 | 87 | 62 | 40 | 17 | 11.9 | A | Y | N | N | 1 |
| **P24** | HBV | Y | 1.4 | 21 | 25 | 40 | 17 | 12.4 | A | N | N | N | 1 |
| **P25** | HBV | Y | 277 | 41 | 46 | 40 | 8 | 12.6 | A | Y | Y | N | 1 |
| **P26** | HBV | Y | 3.7 | 15 | 27 | 40 | 15 | 10.8 | A | N | N | N | 1 |

**Supplementary Table 2: Detailed information of the preoperative status of HCC patients (Continued)**

| **Subjects** | **HBV/HCV** | **Cirrhosis** | **AFP**  **(ng/ml)** | **ALT**  **(U/L)** | **AST**  **(U/L)** | **Albumin**  **(g/L)** | **TBIL**  **(μmol/L)** | **PT**  **(s)** | **C-P Class** | **MVI** | **Tumor size**  **≥5cm** | **Tumor**  **thrombus** | **Tumor**  **number** |
| --- | --- | --- | --- | --- | --- | --- | --- | --- | --- | --- | --- | --- | --- |
| **P27** | NA | Y | 38 | 28 | 42 | 40 | 6.9 | 11.0 | A | N | N | N | 1 |
| **P28** | HBV | Y | 52.8 | 28 | 26 | 43 | 14 | 10.0 | A | Y | Y | N | 1 |
| **P29** | HBV | Y | 3.5 | 15 | 62 | 44 | 13 | 10.5 | A | Y | Y | N | 1 |
| **P30** | HBV | N | ＞1000 | 21 | 33 | 41 | 22 | 12.6 | A | Y | N | N | 1 |
| **P31** | HBV | Y | 99.5 | 45 | 39 | 43 | 14 | 11.6 | A | N | Y | N | 2 |
| **P32** | HBV | Y | 52.9 | 28 | 33 | 39 | 42 | 15.2 | A | Y | N | N | 1 |
| **P33** | HBV | Y | ＞1000 | 32 | 59 | 30 | 44 | 12.7 | A | Y | N | N | 1 |
| **P34** | HBV | N | 31.1 | 7.5 | 29 | 40 | 27 | 11.9 | A | N | Y | N | 1 |
| **P35** | HBV | N | ＞1000 | 112 | 225 | 30 | 25 | 12.5 | A | N | N | N | 1 |
| **P36** | HBV | N | ＜1.3 | 15 | 25 | 47 | 21 | 11.5 | A | N | Y | N | 1 |
| **P37** | HBV | Y | 20.4 | 34 | 34 | 44 | 22 | 11.8 | A | N | N | Y | 1 |
| **P38** | HBV | N | 432.8 | 22 | 43 | 43 | 17 | 10.9 | A | N | Y | N | 1 |
| **P39** | HBV | N | 2.4 | 23 | 24 | 46 | 16 | 10.4 | A | N | N | N | 1 |
| **P40** | HBV | N | ＜1.3 | 16 | 32 | 37 | 19 | 12.6 | A | N | Y | Y | 1 |

Abbreviations: M: Male; F: Female; HBV, hepatitis B virus; HCV, hepatitis C virus; MVI: microvascular invasion; AFP, alpha-fetoprotein; ALT, alanine aminotransferase; AST, aspartate aminotransferase; TBIL: total bilirubin; PT: prothrombin time; C-P Class: Child-Pugh Class; Y: Yes; N, No.
